# *In vivo* Tracking of Dendritic Cell using MRI Reporter Gene, Ferritin

**DOI:** 10.1371/journal.pone.0125291

**Published:** 2015-05-20

**Authors:** Hoe Suk Kim, Jisu Woo, Jae Hoon Lee, Hyun Jung Joo, YoonSeok Choi, Hyeonjin Kim, Woo Kyung Moon, Seung Ja Kim

**Affiliations:** 1 Department of Radiology, Seoul National University Hospital, 101 Daehak-ro, Jongno-gu, Seoul, Korea; 2 Department of Biomedical Science, College of Medicine, Seoul National University, 103 Daehak-ro, Jongno-gu, Seoul, Korea; 3 Department of Radiology, Seoul Metropolitan Government Seoul National University, Boramae Medical Center, 20 Boramae-ro, Dongjag-gu, Seoul, Korea; Istituto Superiore di Sanità, ITALY

## Abstract

The noninvasive imaging of dendritic cells (DCs) migrated into lymph nodes (LNs) can provide helpful information on designing DCs-based immunotherapeutic strategies. This study is to investigate the influence of transduction of human ferritin heavy chain (FTH) and green fluorescence protein (GFP) genes on inherent properties of DCs, and the feasibility of FTH as a magnetic resonance imaging (MRI) reporter gene to track DCs migration into LNs. FTH-DCs were established by the introduction of FTH and GFP genes into the DC cell line (DC2.4) using lentivirus. The changes in the rate of MRI signal decay (R_2_*) resulting from FTH transduction were analyzed in cell phantoms as well as popliteal LN of mice after subcutaneous injection of those cells into hind limb foot pad by using a multiple gradient echo sequence on a 9.4 T MR scanner. The transduction of FTH and GFP did not influence the proliferation and migration abilities of DCs. The expression of co-stimulatory molecules (CD40, CD80 and CD86) in FTH-DCs was similar to that of DCs. FTH-DCs exhibited increased iron storage capacity, and displayed a significantly higher transverse relaxation rate (R_2_*) as compared to DCs in phantom. LNs with FTH-DCs exhibited negative contrast, leading to a high R_2_* in both *in vivo* and *ex vivo* T_2_*-weighted images compared to DCs. On histological analysis FTH-DCs migrated to the subcapsular sinus and the T cell zone of LN, where they highly expressed CD25 to bind and stimulate T cells. Our study addresses the feasibility of FTH as an MRI reporter gene to track DCs migration into LNs without alteration of their inherent properties. This study suggests that FTH-based MRI could be a useful technique to longitudinally monitor DCs and evaluate the therapeutic efficacy of DC-based vaccines.

## Introduction

The noninvasive tracking of the migration of dendritic cells (DCs) into the draining lymph node (LN) where DCs activate T cells, is critical to determine the efficacy of DC-based vaccines [1,2]. Magnetic resonance imaging (MRI) is a clinically feasible and acceptable method to noninvasively visualize and follow the real-time migration of transplanted cells with higher accuracy, and to assure the successful delivery of therapeutic cells to the correct target tissue [3]. A clinically approved superparamagnetic iron oxides (SPIOs) such as Feridex and Resovist has been safely used for the evaluation of *in vivo* DC migration without the alteration of characteristics of the DCs [4,5]. However, the application of SPIOs is limited due to the problem of MRI signal loss such as active ejection of the SPIOs by DCs and death of SPIO-labeled cells [6]. In addition, the SPIOs released from labeled cells can be taken up by other cells or deposited into extracellular structure, leading to changes in MRI signal [6]. Therefore, in order to accurately evaluate the transplanted cell therapy using SPIOs, it is very important to distinguish the specific signal from the cells labeled with SPIOs. Introduction of an imaging reporter gene into cells makes it possible to obtain such specific signal solely from the transplanted cells *in vivo*. Ferritin, a major intracellular iron storage protein, has been known as an endogenous MRI reporter [7]. Stable transduction of ferritin heavy chain (FTH) which stores iron in a soluble, non-toxic form, has recently been applied to track diverse cells, evaluate the therapeutic effect and quantify transplanted cells non-invasively [8–12].

In order to assess MRI and optical imaging of DCs migration into LNs, we here established FTH-DCs transduced with imaging reporter genes, FTH and green fluorescence protein (GFP), using lentivirus system. *In vitro* and *in vivo* MRIs of FTH-DCs were performed using a 9.4 Tesla (T) scanner and an optical imaging system. We here can noninvasively and longitudinally monitor FTH-DCs migration into the popliteal LNs in mice by using MRI. Our results suggest that assessment by MRI of FTH-transduced DCs would be a useful tool to maximize and control the effects of DCs-based immunotherapy.

## Materials and Methods

### DC culture and transduction of reporter genes

DC2.4 cell lines were kindly provided by Dr. K.L Rock (Dana Farber Cancer Institute, Boston, MA) [30]. DC2.4 cells were cultured in RPMI supplemented with 10% fetal bovine serum, 1% penicillin-streptomycin-glutamine, 1% non-essential amino acids, 1% HEPES buffer and 55 μM 2-mercaptoethanol in a 5% CO_2_ incubator at 37°C.

FTH-DCs expressing myc-tagged human FTH and GFP were generated [9]. In brief, DC2.4 cells were transduced with lentivirus (LentiM1.41) for 72 h. Then, GFP-positive cells were sorted using a FACSCalibur flow cytometer (BD Biosciences, Franklin Lakes, NJ, USA) equipped with a 530-nm filter (bandwidth, ± 15 nm), a 585-nm filter (bandwidth, ± 21 nm), and analyzed using a CellQuest software (BD Biosciences). The sorted FTH-DCs were placed in a 96 well plate by the limiting dilution method to create clones from single FTH-DCs, and productive colonies were selected and used for all *in vitro* and *in vivo* studies.

### Immunostaining and Western blot

To evaluate FTH expression in FTH-DCs, cells were cultured on eight-well chamber slides, and rinsed in phosphate buffered saline (PBS) followed by fixation with 2% paraformaldehyde (PFA) for 30 min at 4°C. The fixed cells were incubated with primary antibodies directed against myc and GFP (Santa Cruz Biotechnology, Santa Cruz, CA, USA). The staining was visualized using secondary antibodies conjugated to Alexa 488 (green) and Alexa 594 (red) (Invitrogen, Cergy Pontoise, France). Images of immunostained cells were acquired with a fluorescence microscopy (Leica, Wetzlar, Germany) equipped with a CCD camera. Additionally, the expression of transgene, FTH, was assessed using Western blot. Cells were lysed in RIPA buffer containing a protease inhibitor cocktail (Sigma), and the proteins were separated by SDS-PAGE and transferred to nitrocellulose membranes. The membranes were blocked with 5% non-fat milk in Tris-buffered saline, and incubated with primary antibodies against myc (Santa Cruz Biotechnology) overnight at 4°C and then with horseradish peroxidase-conjugated secondary antibody (Santa Cruz Biotechnology) at room temperature for 30 min. The blots were developed using Enhanced Chemiluminescence Reagents (Amersham Biosciences, Piscataway, NJ, USA).

### *In vitro* Proliferation and migration assays

To examine the cell proliferation activity of DCs and FTH-DCs, cells were seeded in 96-well plates at 10^4^ per well, further cultured for 24 h, 48 h and 72 h, and assessed using a standard 3-,5-diphenyltetrazolium bromide (MTT) assay. The proliferative activity was expressed as the relative ratio at the seeding day.

To assess the migratory ability of DCs and FTH-DCs, cells were stimulated with TNF-α (20 ng/mL, Sigma) and IFN-γ (20 ng/mL, R&D Systems, Minneapolis, MN, USA) for 24 h, andplated into the upper chambers of a trans-well plate (8.0 μm pore size; Corning, Lowell, MA, USA). The lower chambers were filled with 600 μL serum-free medium supplemented with CCL19 (250 ng/mL, R&D Systems) and CCL21 (250 ng/mL, R&D Systems). Cells were incubated for 24 h at 37°C in 5% CO_2_. The migrated cells in the bottom chamber were stained with 4', 6-diamidino-2-phenylindole (DAPI). Images of DAPI-stained slides were acquired with a Leica microscope and recorded with a high-resolution DC300 Leica digital camera (Leica). Five fields were randomly selected and the DAPI-positive cells were quantified as the percentage of area using Leica Qwin image software program.

### RT-PCR

To investigate whether the expression of a stem cell marker is altered in FTH-hMSCs, RT-PCR was performed. Total RNA was isolated from FTH-hMSCs and hMSCs using TRIzol Reagent (Invitrogen, Carlsbad, CA) and cDNA was produced by using SuperScript II reverse transcriptase (Invitrogen). The gene-specific primers used to determine the expression levels of mouse ferritin heavy chain (mFTH), mouse transferrin (mTf) and mouse transferrin receptor (mTfR) and C-C chemokine receptor type-7 (CCR-7) are described in Table 1. The band intensities of the PCR products were calculated using a densitometer (VDS analyzer; Pharmacia Biotech, Uppsala, Sweden) and target mRNA levels were normalized to β-actin levels, which served as an internal control.

**Table 1:** Gene-specific primers used for RT-PCR.

Gene name	Primer sequence (5’-3’)	Size
mouse ferritin heavy chain (mFTH)	Forward: CTGGAACTGCACAAACTGGCReverse: CTCTCATCACCGTGTCCCAG	199
mouse transferrin (mTf)	Forward: CTGTGTCTGGCTGTCCCTGReverse: GACACAACTGCCCGAGAAGA	488
mouse transferrin receptor (mTfR)	Forward: GCACGAGGGTAGAGGCGReverse: TAGCCCAGGTAGCCACTCAT	309
mouse beta-actin(β-actin)	Forward: TTCCTGGGCATGGAGTCCTGReverse: CGCCTAGAAGCATTTGCGGT	335

### Flow cytometry analysis

To analyze the expression of co-stimulatory molecules, CD40, CD80 and CD86 in DCs and FTH-DCs, cells were pulsed with 1μg/ml with lipopolysaccharide (LPS) for 24 h, collected and washed twice with ice-cold PBS containing 1% BSA and 1 mM EDTA. After cells were incubated with anti-CD40-PE, anti-CD80-FITC and anti-CD86-FITC antibodies (BD Biosciences) for 30 min at 4°C, cell-associated fluorescence was measured using a FACSCalibur flow cytometer (BD Biosciences). Data were analyzed with CellQuest v3.3 software (BD Biosciences).

### Prussian blue staining and measurement of cellular iron levels

Iron deposits of DCs and FTH-DCs were examined by Prussian blue staining and cellular iron measurement. In brief, cells were incubated in medium supplemented with ferric ammonium citrate (FAC, 25 μM and 250 μM) for 10 h. After washing with PBS twice cells were fixed with 2% PFA at 4°C for 1 hour and incubated with staining solution by mixing equal volumes of 5% potassium ferrocyanide and 5% hydrochloric acid for 30 min. Counterstaining was performed with nuclear fast red for 5 min.

Cellular iron levels were measured using the Total Iron Reagent Set (Pointe Scientific, Canton, MI, USA). Briefly, cells were incubated in 25 μM and 250 μM FAC-supplemented medium for 10–24 h, rinsed with PBS, and enumerated. Cells were collected, resuspended in 6N HCl solution, and incubated at 70°C for 30 min. According to the manufacturer's recommendations, total iron levels were measured, and average iron concentrations in cells were calculated by dividing total mean values by cell number.

### Animal study and cell transplantation

C57BL/6 mice were housed in specific pathogen-free conditions, and all animal experiments were approved by the Seoul National University Hospital Biomedical Research Institute Animal Care and Use Committee (IACUC). A total of 20 male mice were used in MRI and histological studies. Twenty-four h before the cell transplantation, 6-7-week old mice were subcutaneously injected with TNF-α (40 ng) to activate vascular and lymphatic endothelial cells. Mice were anesthetized with an intramuscular injection of a zoletil (7–10 mg/kg). DCs and FTH-DCs were treated with TNF-α (20 ng/mL) and IFN-γ (20 ng/mL) for 24 h. A total of 1 x 10^7^ DCs and FTH-DCs in 50 μl PBS were injected subcutaneously into the right and left hind footpads of mice and MRI study and histological analysis were performed.

### *In vivo* and *ex vivo* fluorescence imaging of FTH-DCs-injected mice

1x10^7^FTH-DCs were intraperitoneally injected of mice. At 3 day and 5 day post-injection, mice were anesthetized with an intramuscular injection of a zoletil, the abdomen of mice was opened, and the organs were excised. *In vivo* and *ex vivo* fluorescence images were obtained at using Maestro imaging system (CRi, Woburn, MA, USA) and the spectral fluorescence images consist of autofluorescence spectra and the spectra from GFP, which were then unmixed, based on their spectral patterns using Maestro software (CRi).

### MRI studies and data analysis

To evaluate whether FTH transduction can generate T_2_* contrast *in vitro*, *in vivo* and *ex vivo*, MRI studies were performed on a 9.4T animal MR scanner (Agilent, Santa Clara, CA, USA). A transmit-only volume coil (Agilent) and a phased-array 4-channel surface coil (Agilent) were used for RF excitation and signal reception, respectively. For *in vitro* MRI of cell phantoms, cells were cultured in medium with or without iron supplement of 25 μM and 250 μM FAC for 10–24 h, harvested and enumerated by direct viable count. Cells were resuspended in 0.7% agarose and transferred to 200 μl tubes. To evaluate the detection sensitivity for MRI, FTH-DCs were treated with 25 μM FAC for 10 h and cell phantoms consisting of 1x10^5^, 1x10^6^, 5x10^6^, and 1x10^7^ FTH-DCs were prepared. For *ex vivo* MRI of excised popliteal LNs, LNs were harvested immediately after *in vivo* MRI studies. T_2_*-weighted images of cell phantoms and excised LNs were acquired using a multiple gradient echo sequence. The sequence parameters were as follows: repetition time (TR) = 3000 ms, echo times (TEs) = 2.0–20.0 ms with a step size of 2.0 ms (i.e., 10 point R_2_* mapping), flip angle (FA) = 50^°^, field-of-view (FOV) = 39 x 35 mm^2^, matrix size = 128 x 128, number of slices = 5 (gap = 0), slice thickness (TH) = 1.0 mm, number of examinations (NEX) = 1, and receiver bandwidth = 50 kHz.

Mice in the prone position were placed on the warm plate in the MR scanner. Before and at 48 h after transplantation of DCs and FTH-DCs, *in vivo* T_2_*-weighted images of the popliteal LNs of mice were acquired in the axial plane. An 8-point R_2_* mapping was performed using a fat-suppressed multiple gradient echo sequence. The imaging parameters were as follows: TR = 3000 ms, TE = 3.2, 7.65, 12.1, 16.55, 21.0, 25.45, 29.9, 34.35 ms, FA = 90^°^, FOV = 35 x 33 mm^2^, matrix size = 256 x 256, number of slices = 8 (gap = 0), TH = 0.5 mm, NEX = 3, and receiver bandwidth = 50 kHz.

All post-data processing was performed using MATLAB^TM^ (v.7.13; Mathworks Inc.**,** Natick, MA, USA). For each slice, regions of interest (ROIs) were manually defined on the images acquired at the shortest TE, and R_2_* values were estimated pixel by pixel assuming a single exponential decay of signal across the images at multiple TEs. Then, a mean R_2_* value was estimated from all pixels in the ROIs across all slices, and used in the final statistical analysis. R_2_* histograms were also generated from these pixels for comparison between DCs and FTH-DCs.

### Histological analyses

Popliteal LNs were isolated from mice at 48 h post-injection immediately after in vivo MRI studies. The removed LNs were fixed in 4% PFA and laid in 30% sucrose solution (Sigma) overnight. Tissues were embedded in Optimal Cutting Temperature (OCT; Sakura Finetek, Torrance, CA, USA) and sectioned at a thickness of 5 μm (Leica). Cryo-sectioned LNs were prepared, and Prussian blue staining, hematoxylin and eosin (H&E) staining and immunofluorescence staining using anti-GFP, anti-myc, and anti-CD25 antibodies (Novus Biologicals, Littleton, CO, USA) were performed. Images were captured and analyzed using the Leica Qwin program.

### Statistical analyses

All data are presented as mean ± standard deviation. Data were statistically processed using one-way ANOVA followed by the Student-Newman-Keuls test. For all tests, a *p-*value of less than 0.05 was considered to indicate statistical significance.

## Results

### FTH transduction did not alter the proliferation and migration ability of DCs and the presentation of co-stimulatory molecules on DCs

**Fig 1:**
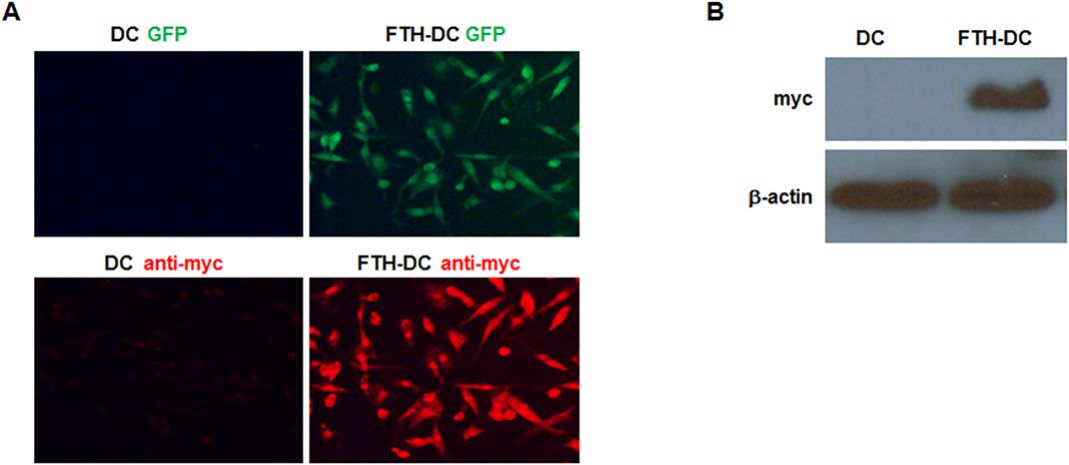
Analysis of dendritic cell (DC) transduced with myc-tagged human ferritin heavy chain (FTH) and green fluorescence prtein (GFP) using lentivirus. (A) Representative GFP fluorescence image (green) and FTH immunofluorescence image (red) detected with anti-myc antibody in DCs and FTH-DCs. (B) Representative Western blots for FTH from whole cell lysate of DCs and FTH-DCs detected by using anti-myc antibody.

DC2.4 cells were transduced with GFP and myc-tagged FTH genes using lentivirus. Abundant expression of GFP proteins in FTH-DCs were observed under the microscope (Fig 1A). The high expression of myc-tagged FTH transgene in FTH-DCs was detected by immunostaining and Western blot using anti-myc antibody (Fig 1A and 1B).

To further investigate the changes of mouse FTH (mFTH), mouse transferrin receptor (mTfR) and mouse transferrin (mTf), which were involved in the control of cellular iron metabolism, RT-PCR were assessed in the DCs and FTH-DCs treated with or without 25 μM and 250 μM FAC. As shown in S2 Fig, the expression levels of mFTH and mTfR were similar between DCs and FTH-DCs. mFTH was slightly increased whereas mTfR was decreased dose-dependently in both cells treated with an increasing FAC (S2 Fig). The expression level of mTf, which are iron-binding blood plasma glycoproteins to control the level of free iron in biological fluids was lower in FTH-DCs than DCs (S2 Fig).

**Fig 2:**
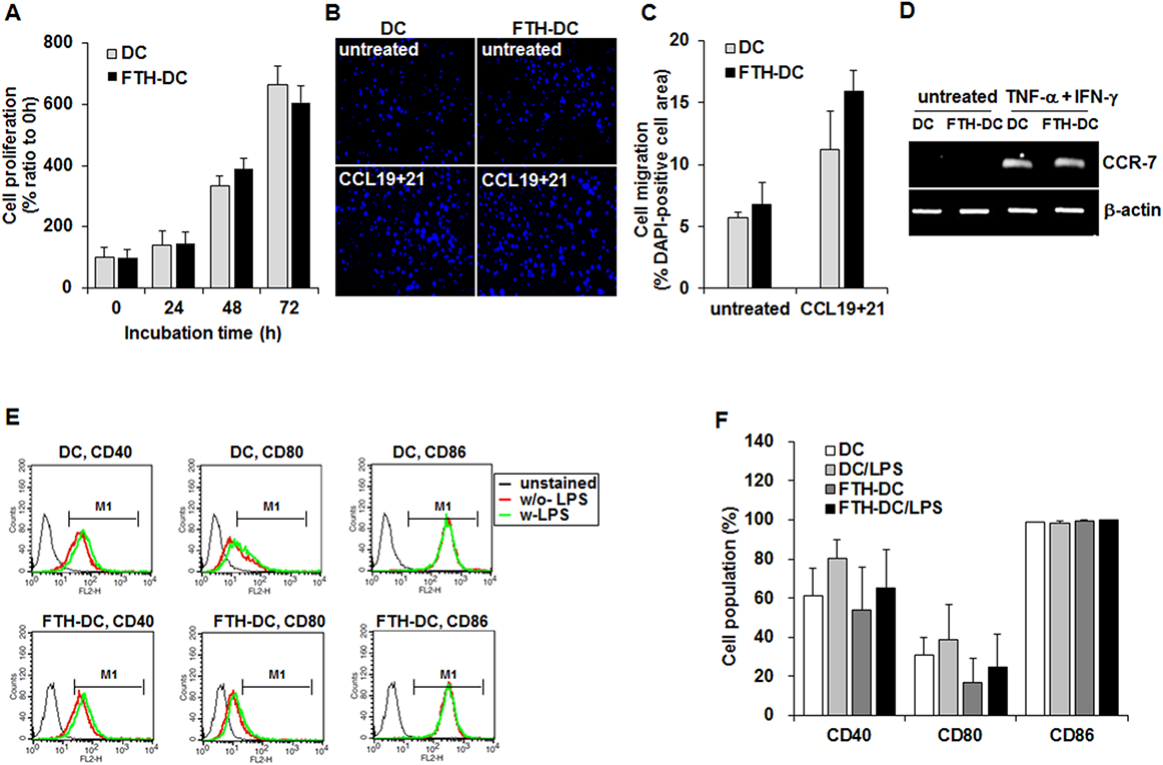
Analyses of proliferation and migration activities and co-stimulatory molecules expressions in dendritic cell (DC) and human ferritin heavy chain-transduced DC (FTH-DC). (A) A standard 3-,5-diphenyltetrazolium bromide (MTT) assay for proliferation activity of DCs and FTH-DCs cultured for 24 h, 48 h and 72 h. (B and C) Trans-well assay for migration abilities of DCs and FTH-DCs incubated with TNF-α (20 ng/mL) and IFN-γ (20 ng/mL) in the presence or absence of CCL19 and CCL21 for 24 h. Representative fluorescent image of nuclear stained with diamidino-2-phenylindole (DAPI) in DCs and FTH-DCs that migrated to the lower chamber. (D) RT-PCR analysis of C-C chemokine receptor type-7 (CCR-7) in DCs and FTH-DCs. Both DCs and FTH-DCs, which were incubated in the medium supplemented with TNF-α (20 ng/mL) and IFN-γ (20 ng/mL) for 24 h, highly expressed the CCR-7 as compared to untreated cells. (E and F) Representative flow cytometric analysis of co-stimulatory molecules such as CD40, CD80 and CD86 in DCs and FTH-DCs treated with or without LPS (100 ng/mL) for 24 h. Flow cytometric results obtained from 3 independent experiments. All data are presented as the mean ± standard deviations of at least three independent experiments. *, *p* ≤0.05.

The cellular proliferation and migratory ability resulting from introduction of transgenes in FTH-DCs were evaluated by a MTT assay and *in vitro* migration assay. Introduction of FTH and GFP transgenes did not influence cell proliferation (Fig 2A, *p*>0.1, DC versus FTH-DC). However, DCs and FTH-DCs did not proliferate at 48h after the treatment with with TNF-α (20 ng/mL) and IFN-γ (20 ng/mL). Both DCs and FTH-DCs stimulated with TNF-α and IFN-γ in the presence of CCL-19 and CCL-21 exhibited enhanced migratory ability compared to untreated control cells (Fig 2B) and there was no statistically significant difference in migration ability between DCs and FTH-DCs (Fig 2C). Here we additionally performed the RT-PCR to evaluate the expression levels of C-C chemokine receptor type-7 (CCR-7), which is the gate-keeper to regulate the mobilization of DCs from the periphery to the LNs, in FTH-DCs treated with TNF-α (20 ng/mL) and IFN-γ (20 ng/mL) for 24 h. CCR-7 was upregulated in TNF-α and IFN-γ treated-DCs and FTH-DCs compared to untreated cells (Fig 2D). Next, we examined whether the introduction of transgenes, FTH and GFP with lentivirus alters the expression levels of co-stimulatory molecules on the surface of DCs. Fig 2E is representative flow cytometry data of CD40, CD80 and CD86, which are important for effective T cell activation, in DCs and FTH-DCs. The expression levels of CD40, CD80 and CD86 were increased by LPS treatment in both DCs and FTH-DCs, but not affected by FTH and GFP transgenes (Fig 2F, *p*>0.05, DC versus FTH-DC and DC/LPS versus FTH-DC/LPS).

### Ferritin transduction increased the cellular iron storage and caused T_2_ contrast enhancement in MR images of cell phantom

**Fig 3:**
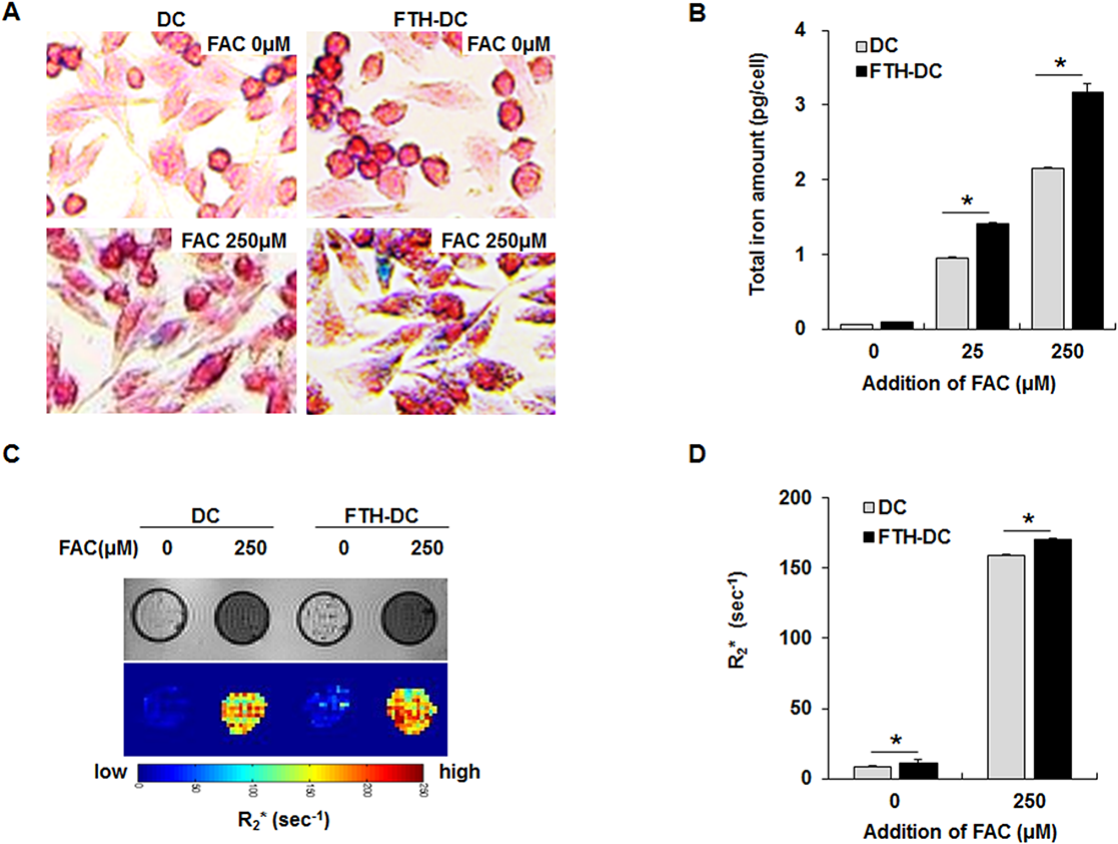
Cellular iron staining, iron amount measurement and in vitro MRI analysis of dendritic cell (DC) and human ferritin heavy chain-transduced DC (FTH-DC). (A) Representative prussian blue staining of cellular iron in DCs and FTH-DCs incubated with or without 250 μM ferric ammonium citrate (FAC) for 24 h. (B) Average cellular iron amount measured from DCs and FTH-DCs incubated with 25 μM and 250 μM FAC. (C) Representative T_2_*-weighted images and color-coded of the R_2_* values in the DCs and FTH-DCs phantoms. (D) Average R_2_* values measured from DCs and FTH-DCs phantoms. All data are presented as the mean ± standard deviations of at least three independent experiments. *, *p* ≤0.05.

The iron storage capacity of DCs and FTH-DCs incubated with or without iron supplement, FAC was investigated. The irons stained with Prussian blue were observed as blue dots in the cytosol of DCs and FTH-DCs incubated with 250 μM FAC for 24 h (Fig 3A). However blue dots were not observed in both DCs and FTH-DCs incubated with 25 μM FAC (data not shown). Iron levels measured from DCs and FTH-DCs cultured without treatment of FAC were 0.06 ± 0.001 and 0.09 ± 0.004 pg/cell, respectively (*p* = 0.08). In the presence of 25 μM FAC for 10 h, cellular iron levels of DCs and FTH-DCs were 0.95 ± 0.02 pg/cell and 1.42 ± 0.021 pg/cell, respectively (*p* = 0.016). As the 250 μM FAC was added for 10 h, cellular iron levels increased in both DCs and FTH-DCs were 2.15 ± 0.033 pg/cell and 3.18 ± 0.15 pg/cell, respectively (*p<*0.001). The iron storage capacity was further enhanced in FTH-DCs compared to DCs (Fig 3B). To investigate the changes in the rate of MRI signal decay (R_2_*) by the expression of FTH transgenes as endogenous contrast agent, *in vitro* MRI analysis of cell phantom was performed. After adding FAC, the areas with hypointense signal in the T_2_*-weighted images were increased for both phantoms containing 5x10^7^ cells (Fig 3C, upper). The color-coded R_2_* maps showed that FTH-DCs have higher R_2_* than DCs (Fig 3C, lower). The mean R_2_* values of DCs and FTH-DCs untreated with FAC were 8.84 ± 0.04 sec^-1^ and 11.03 ± 0.05 sec^-1^ (DCs versus FTH-DCs, *p* = 0.043), and the mean R_2_* values of DCs and FTH-DCs treated with 250 μM FAC were 159.36 ± 1.01 sec^-1^ and 170.48 ± 1.44 sec^-1^, respectively (FAC-treated DCs versus FAC-treated FTH-DCs, *p*<0.001) (Fig 3D).

The mean R_2_* values were significantly higher in in FTH-DCs as compared to DCs. We conducted an additional *in vitro* MRI of cell phantoms by varying the cell numbers and measured the R_2_ values to evaluate the detection sensitivity for FTH-DCs. The R_2_ values of 1×10^5^, 1×10^6^, 5×10^6^, and 1×10^7^ FTH-DCs incubated with 25 μM FAC were 8.04 ± 0.02 sec^-1^, 8.43 ± 0.02 sec^-1^, 9.50 ± 0.07 sec^-1^ and 11.03 ± 0.05 sec^-1^ respectively (S1 Fig)

### Ferritin-transduced DCs were noninvasively monitored in popliteal LNs of mice using a 9.4T MR scanner

**Fig 4:**
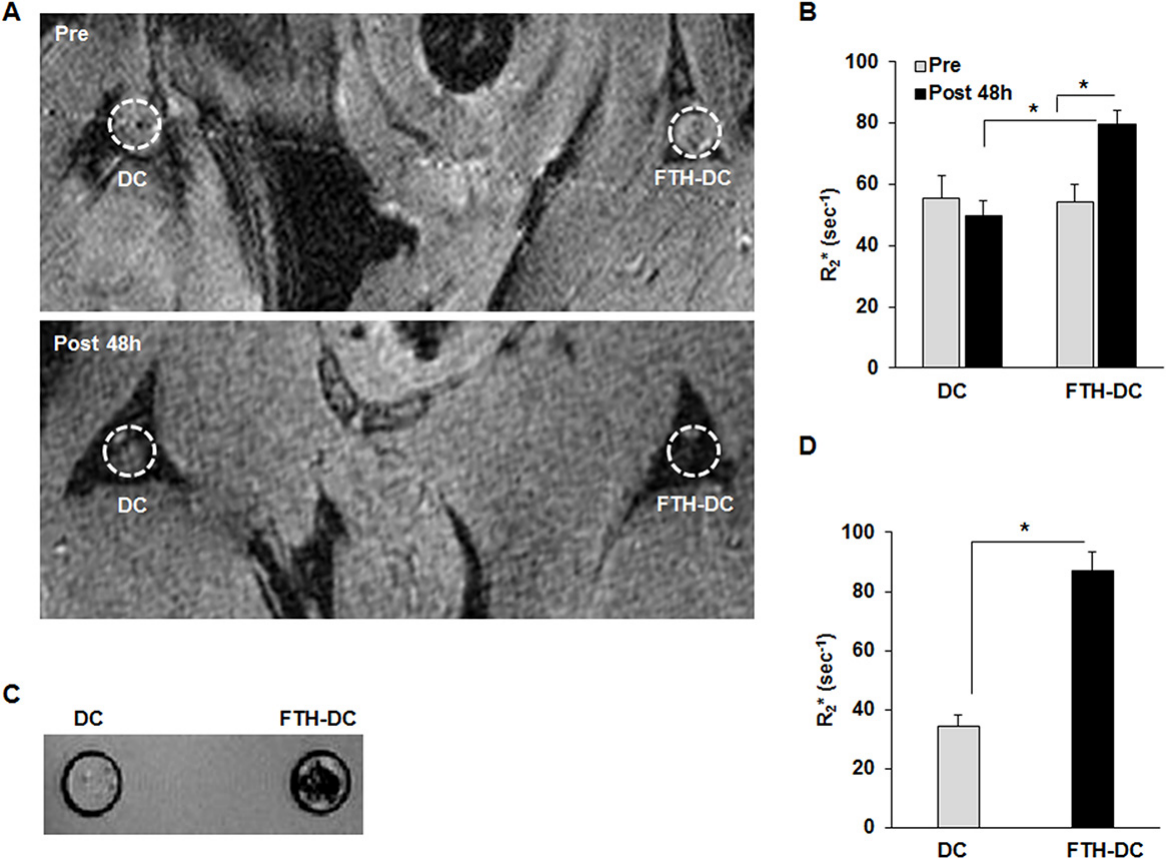
*in vivo* and *ex vivo* MRI of popliteal lymph nodes (LNs) of mouse injected with dendritic cell (DC) and ferritin heavy chain-transduced DC (FTH-DC). (A) Representative *in vivo* T_2_*-weighted images of popliteal LNs (circle) of mouse before and at 48 h after injection of 1x10^7^ DCs and FTH-DCs. (B) Average R_2_* values measured from *in vivo* popliteal LNs with DCs and FTH-DCs. (C) Representative *ex vivo* T_2_*-weighted images of popliteal LNs isolated from mouse at 48 h after injection of DCs and FTH-DCs. (D) Average R_2_* values measured from *ex vivo* popliteal LNs with DCs and FTH-DCs. All data are presented as the mean ± standard deviations of at least three independent experiments. *, *p*≤0.05

To investigate whether the expression of FTH transgenes would allow for non-invasive detection of DC migration into draining popliteal LNs using MRI, *In vivo* MR images were acquired before and after the injection of DCs and FTH-DCs into the footpad of mice. FTH-DCs resulted in signal loss on the fat-suppressed multi-slice T_2_*-weighted images of popliteal LNs of mice at 48 h post-injection while DCs did not lead to such signal changes (Fig 4A). The signal changes were further analyzed quantitatively with R_2_* mapping. For the quantitative analysis of the signal changes from each popliteal LN, the mean R_2_* value was analyzed from 2–3 image slices. The mean R_2_* values of the left (Pre-DC) and the right (Pre-FTH-DC) popliteal LNs before the transplantation of cells were 55.69 ± 7.0 sec^-1^ and 54.31 ± 5.87 sec^-1^, respectively (Fig 4B). At 48 h post injection, the mean R_2_* value of the popliteal LNs with FTH-DC significantly changed to 79.52± 3.18 sec^-1^ (pre-FTH-DC versus post-FTH-DC, *p* = 0.001) and was higher than that of the popliteal LNs with DC (49.73 ± 4.74 sec^-1^) (Fig 4B, post-DCs versus post- FTH-DCs, *p* = 0.001). We further analyzed the *ex vivo* MRI of the isolated popliteal LNs. A large amount of signal loss was clearly observed in the *ex vivo* T_2_*-weighted image of the popliteal LNs with FTH-DC with respect to that with DC (Fig 4C). The mean R_2_* values of DC and FTH-DC measured *ex vivo* were 34.28 ± 10.05 and 87.01 ± 15.65 sec^-1^, respectively (Fig 4D, *p*<0.001).

### FTH-DCs migrated into draining LNs expressed the co-stimulatory and binding molecules for activating and binding T-cells

**Fig 5:**
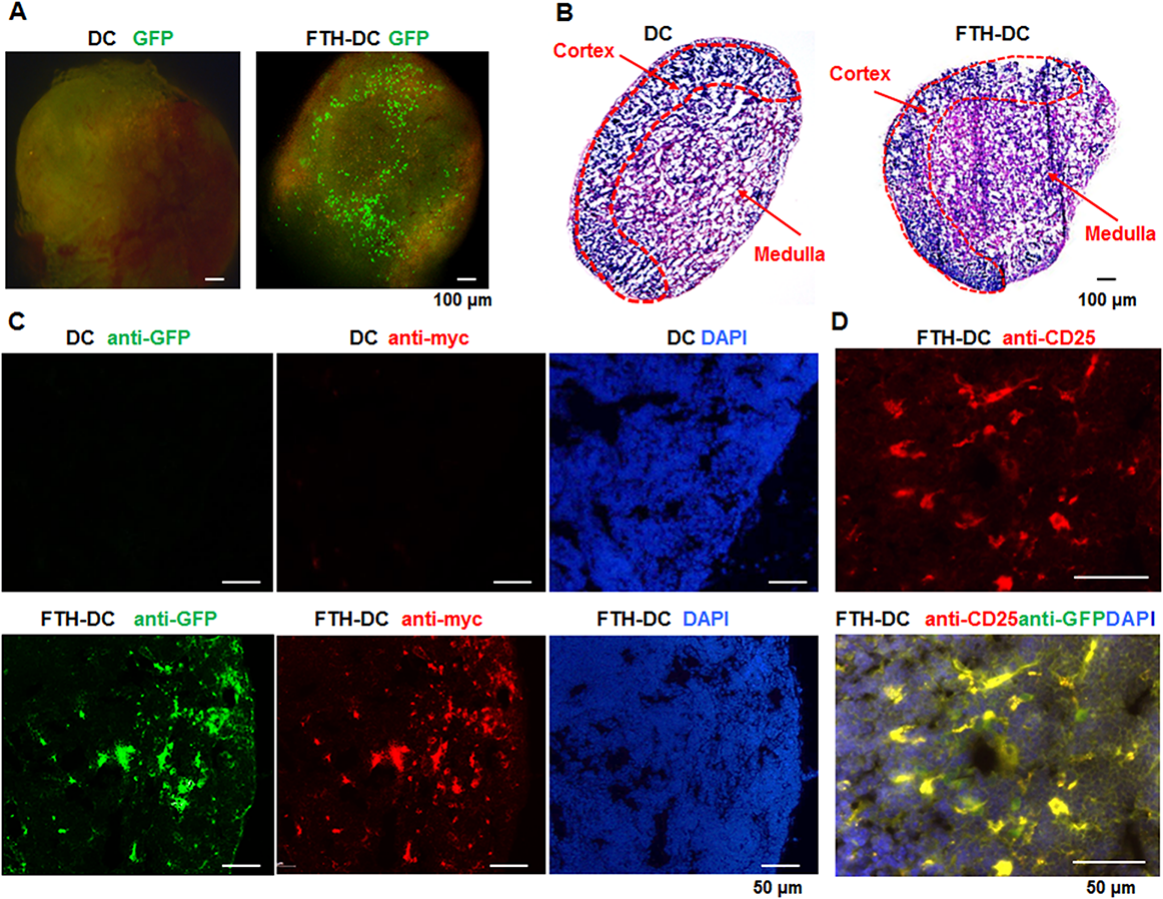
Histological analysis of cryosectioned popliteal lymph nodes (LNs) with dendritic cell (DC) and ferritin heavy chain-transduced DC (FTH-DC). (A) Representative GFP fluorescence images of popliteal LNs isolated from mouse at 48 h after injection of DCs and FTH-DCs. (B) Representative hematoxylin and eosin staining of cryosectioned popliteal LNs with DCs and FTH-DCs. (C) Immunofluorescence images of GFP (green) and FTH (red) detected with anti-GFP and anti-myc antibodies in LNs with DCs and FTH-DCs. (D) Merge images (yellow) of co-immunofluorescence staining of activation marker, CD25 (red) and GFP (green) detected with anti-CD25 and anti-GFP antibodies in FTH-DCs of cryosectioned LNs. Nuclear was stained with diamidino-2-phenylindole (DAPI, blue).

At 48 h after the injection of the cells, *ex vivo* fluorescence images of the isolated popliteal LNs showed the expression of GFP transgenes from FTH-DCs under the fluorescent inverted microscope (Fig 5A). The histological examinations of the popliteal LN cryosections of the DCs and FTH-DCs groups were assessed by H&E staining. The cortex that is the bulk of the lymphatic tissue and the medulla that is less densely packed were easily visible in the slides of H&E staining. H&E staining showed no histologically architectural differences of cortex and medulla between DCs-LN and FTH-DCs-LN (Fig 5B). The existence of FTH-DCs in the popliteal LNs was confirmed by the immunostaining of GFP and myc where a large number of FTH-DCs were observed below the LN capsules as well as T cell zone (Fig 5C). We further investigated whether FTH-DCs that did reach the T cell zone of the LN, express CD25 on the cell surface of mature DCs for binding and activating T cells. The concomitant expressions of the activation marker, CD 25, were detected on the same site of the GFP expressed FTH-DCs in the center of LN (Fig 5D).

For the study of the recovery of FTH-DCS in the LN, GFP-positive cells in popliteal LNs isolated from mouse were analyzed by flow cytometry. About 2.45 x 10^4^ GFP-positive cells in popliteal LNs were detected at 48 h post-injection (S3 Fig). To show a more viable option in humans, we investigated whether intraperitoneally injected FTH-DCs still migrate to some other organs first before they reach LN. At 3 and 5 days after intraperitoneal injection of FTH-DCs, GFP fluorescence can be observed in isolated pancreas, mesentery and spleen using the Maestro *in vivo* imaging system. These results demonstrated that FTH-DCs still migrated to other organs and reached at LN in the pancreas or the mesentery as well as the spleen, which is largest lymphatic organ (S5 Fig).

## Discussion

DCs are the most potent antigen-producing cells in the induction of antigen-specific T cell responses, and therefore DCs-based immunotherapy has been studied for the treatment of diverse cancers in preclinical and clinical applications with considerable interest [13,14]. Developing a better means of tracking the migration of DCs to LNs is essential for future advances in manipulating DCs migration for fine-tuning and boosting of immune responses in clinical settings [2,3]. Additionally, since individual patients can drive different immune responses to DCs therapy, monitoring injected DCs in vivo may be of major importance.

Noninvasive molecular imaging techniques including MRI, optical imaging, and nuclear imaging have been suggested to be a significant tool to evaluate the DCs-based immunotherapy by monitoring *in vivo* migration patterns and antigen expressions of *in vitro*-generated DCs [3,15,16]. Intravital microscopy allows visualization of the exact interactions between DCs and T cells in intact LNs [17,18]. So far, many studies have demonstrated the safety of SPIOs as a labeling agent for DCs without causing biological alterations in addition to the capability of the agent in visualizing DCs migration *in vivo* using MRI [5,19–21]. Our previous study demonstrated that MRI technique visualized the enhanced migration ability of SPIO-labeled DCs treated with prostaglandin E2 and provided *in vivo* information regarding the migratory patterns of the injected DCs over the following days [16]. In that study, however, it was challenging to distinguish healthy cells from SPIO-labeled DCs migrated into LN by using MRI. Noninvasive tracking and monitoring of healthy DCs can provide more accurate information in the evaluation of the therapeutic efficacy of DCs-based immunotherapy. Although many groups have demonstrated the feasibility of sequential MRI of diverse cells by utilizing MRI reporter genes, FTH, at high magnetic field [7,9–12], to the best of our knowledge, there has been no trial reported to date to monitor DCs using FTH-based MRI.

Moreover, to maintain durable and efficient antigen expression and presentation in DCs both *in vitro* and *in vivo* is critical for enhancing the activation of cellular immunity and humoral responses [4,22]. Adenoviral vectors have been widely used in gene transfer studies but are relatively inefficient in transduction and transgene expression, resulting in suppressing T cell stimulatory capacity of DC function [23]. Lentiviral vectors have been successfully employed as a gene transfer vehicles and are powerful tools for immunizations, because they are able to transduce both DCs and other antigen producing cells efficiently, resulting in long-term antigen expression and presentation [24,25]. In this study, we have demonstrated that the high and stable expression of FTH and GFP reporter genes by utilizing lentiviral vector did not alter the cell proliferation and migration abilities as well as the phenotypic maturation of DCs. In addition, we demonstrated the feasibility of noninvasive and longitudinal monitoring of DCs migration into LNs using stable transduction of FTH on a 9.4T MR scanner. Our result also provides a good control in the lentivirus model, as transduction/ transfection may vary in different cell types.

DCs efficiently take up exogenous antigens, migrate into the T-cell area of LN and contribute to tailor T cell functions in generating protective immunity or immune tolerance. Interactions between DCs and T cells in the LNs are crucial for initiating cell-mediated adaptive immune responses [26]. Mature DCs with strong stimulatory properties express CD25 on their cell surface for binding T cell, which is the early mechanism before T cells start to express CD25 [27,28]. The mature DCs exhibit high expressions of activation marker molecules such as CD25 as well as co-stimulatory molecules such as CD40, CD80 and CD86 [29]. We here observed that those FTH-DCs migrated into popliteal LNs exhibited T cell activation molecule CD25. Our current result supports that overexpression of the MRI reporter, FTH, does not affect *in vivo* DCs maturation and migration into LNs to prime T cell.

In conclusion, this study is a preclinical trial that monitored DCs by means of MRI reporter genes, FTH transduction. Our study suggests the feasibility of FTH-based MRI to noninvasively image DCs without alteration of innate biological properties, which may aid in optimizing DCs-based immunotherapies on tumor or other diseases. Furthermore, our vector system could offer a new opportunity to simultaneously deliver to DC and overexpress targeted antigen. FTH-based MRI can allow not only for the actual tracking, at least, of healthy DCs, but also for assurance of successful delivery of specific antigen to DCs to improve therapeutic outcomes. Thus FTH-based MRI technique would be a favorable tool for prediction and validation of DC-based therapeutic effect.

## Supporting Information

S1 Fig *In vitro* MRI of cell phantoms at different cell numbers. (A) T_2_-weighted images and color-corded R_2_ map image of phantoms consisting of 0 ~ 1 x 10^7^ FTH-DCs cells treated with 25 μM ferric ammonium citrate (FAC) for 10 h. (B) The plot of transverse relaxation rate (R_2_) measured from FTH-DCs phantoms.
(TIF)

S2 Fig RT-PCR analysis of iron-related genes, mouse ferritin heavy chain (mFTH), mouse transferrin receptor (mTfR) and mouse transferrin (mTf). DCs and FTH-DCs cells incubated for 24 h in the presence of absence of 25 μM or 50 μM ferric ammonium citrate (FAC). The expression levels of mFTH and mTfR were similar between DCs and FTH-DCs. In both cells treated with an increasing FAC, mFTH was slightly increased whereas mTfR was decreased dose-dependently. The expression level of mTf was lower in FTH-DCs than DCs.
(TIF)

S3 Fig The recovery of FTH-DC in the lymph node (LN). Twenty-four h before the cell transplantation, 6-7-week old mice (n = 5) were subcutaneously injected with TNF-α (40 ng). FTH-DCs were incubated in the medium supplemented with TNF-α (20 ng/mL) and IFN-γ (20 ng/mL) for 24 h. A total of 1 x 10^7^ FTH-DCs were injected subcutaneously into the hind footpads of mice. At 48 h after injection, GFP-positive cells in popliteal LNs isolated from mouse were analyzed by flow cytometry. About 2.45 x 10^4^ GFP-positive cells in popliteal LNs were detected.
(TIF)

S4 Fig Fig 2. Analyses of proliferation activities in stimulated DC and FTH-DCs. A standard 3-,5-diphenyltetrazolium bromide (MTT) assay for proliferation activity of DCs and FTH-DCs stimulated by with TNF-α (20 ng/mL) and IFN-γ (20 ng/mL) for 24 h and 48 h.
(TIF)

S5 Fig Images of green fluorescence protein in the isolated organs, pancreas, spleen, mesentery after peritoneal injection of FTH-DC. 6-7-week old mice (n = 5) were intraperitoneally injected with 1x10^7^ FTH-DCs incubated in the medium including TNF-α (20 ng/mL) and IFN-γ (20 ng/mL) for 24 h. A total of 1 x 10^7^ FTH-DCs were injected subcutaneously into the hind footpads of mice. At 3 and 5 days after intraperitoneal injection of FTH-DCs, GFP fluorescence can be observed in isolated pancreas, mesentery and spleen using the Maestro fluorescence imaging system. These results demonstrated that FTH-DCs still migrated to other organs and reached at LN in the pancreas or the mesentery as well as the spleen, which is largest lymphatic organ.
(TIF)
