# Supplementary figures and images for: In vivo Tracking of Dendritic Cell using MRI Reporter Gene, Ferritin
Source: PLoS One. 2015 May 20;10(5):e0125291. doi: 10.1371/journal.pone.0125291 (PMC4439152; doi:10.1371/journal.pone.0125291)

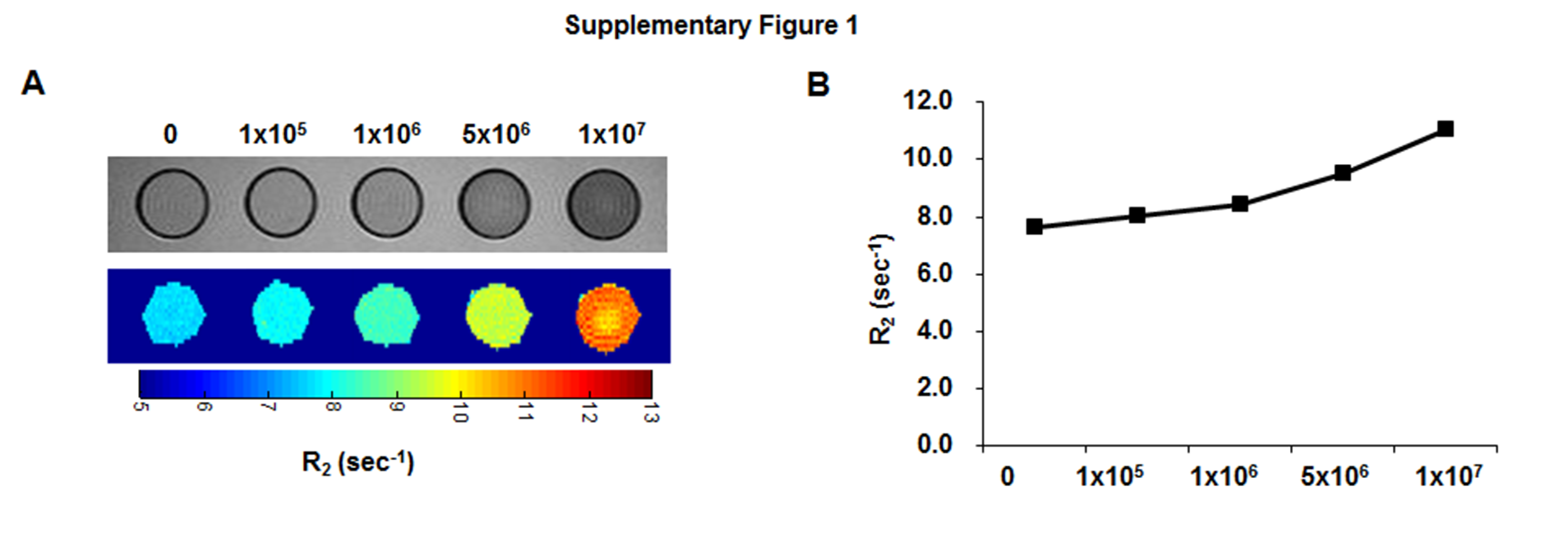

Supplement: S1 Fig — (A) T2-weighted images and color-corded R2 map image of phantoms consisting of 0 ~ 1 x 10^7^ FTH-DCs cells treated with 25 μM ferric ammonium citrate (FAC) for 10 h. (B) The plot of transverse relaxation rate (R2) measured from FTH-DCs phantoms. (TIF) [file pone.0125291.s001.tif]

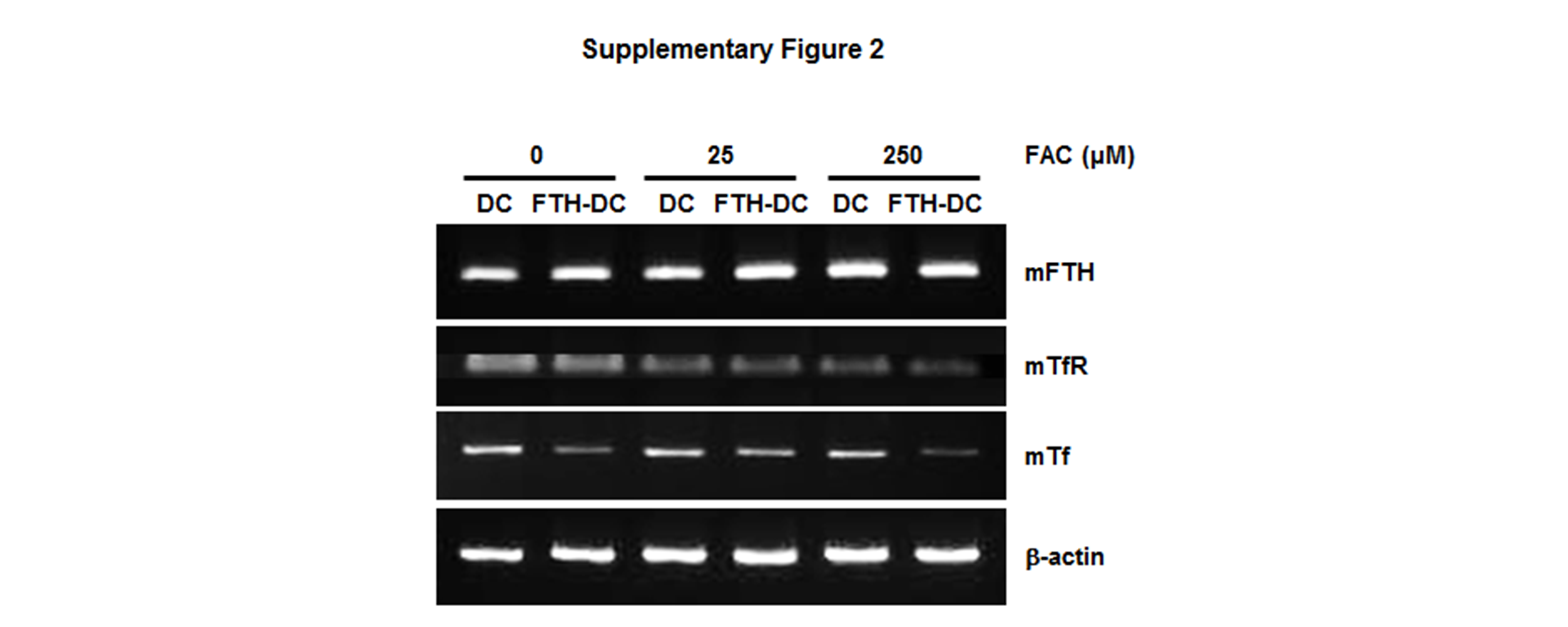

Supplement: S2 Fig — DCs and FTH-DCs cells incubated for 24 h in the presence of absence of 25 μM or 50 μM ferric ammonium citrate (FAC). The expression levels of mFTH and mTfR were similar between DCs and FTH-DCs. In both cells treated with an increasing FAC, mFTH was slightly increased whereas mTfR was decreased dose-dependently. The expression level of mTf was lower in FTH-DCs than DCs. (TIF) [file pone.0125291.s002.tif]

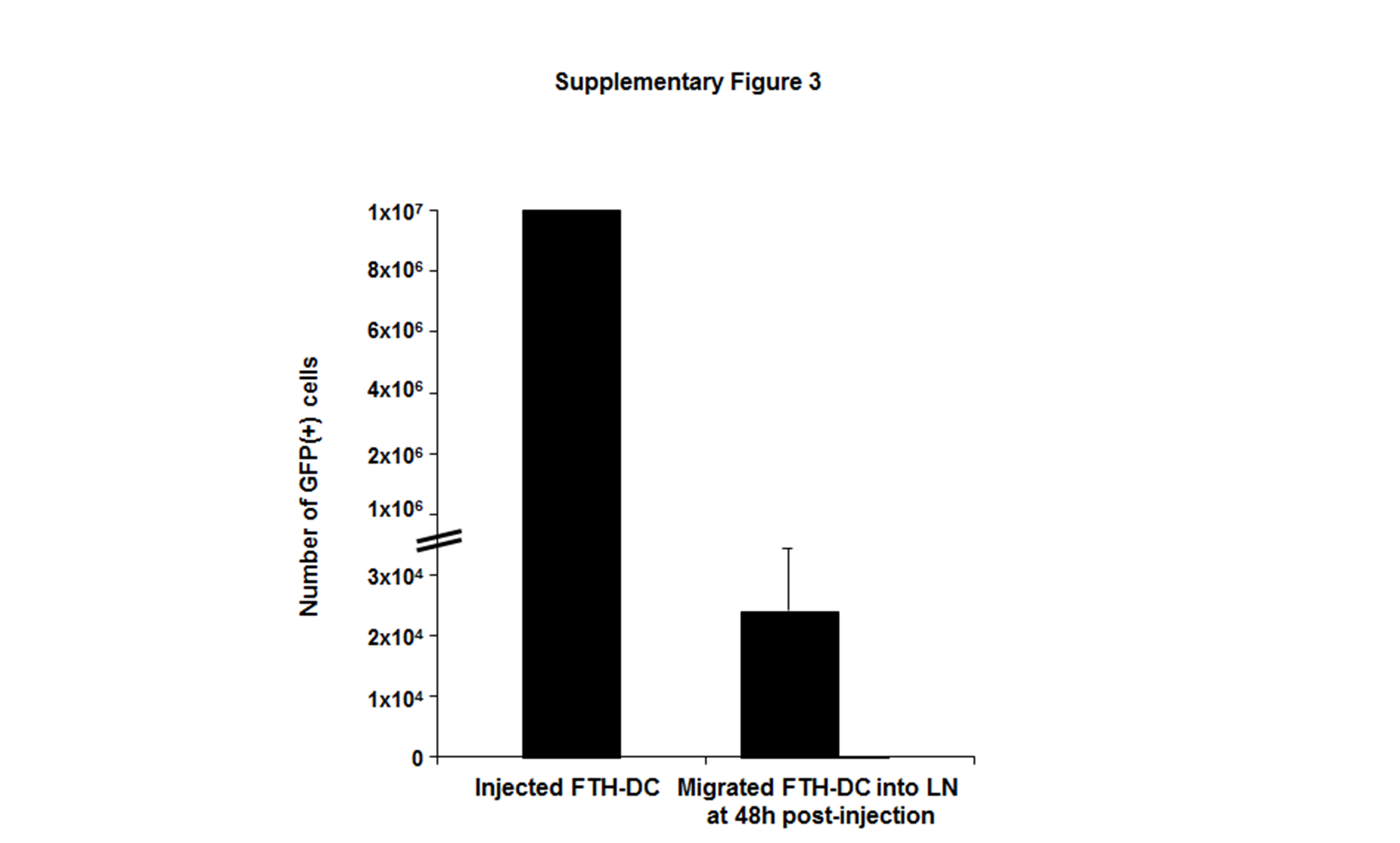

Supplement: S3 Fig — Twenty-four h before the cell transplantation, 6-7-week old mice (n = 5) were subcutaneously injected with TNF-α (40 ng). FTH-DCs were incubated in the medium supplemented with TNF-α (20 ng/mL) and IFN-γ (20 ng/mL) for 24 h. A total of 1 x 10^7^ FTH-DCs were injected subcutaneously into the hind footpads of mice. At 48 h after injection, GFP-positive cells in popliteal LNs isolated from mouse were analyzed by flow cytometry. About 2.45 x 10^4^ GFP-positive cells in popliteal LNs were detected. (TIF) [file pone.0125291.s003.tif]

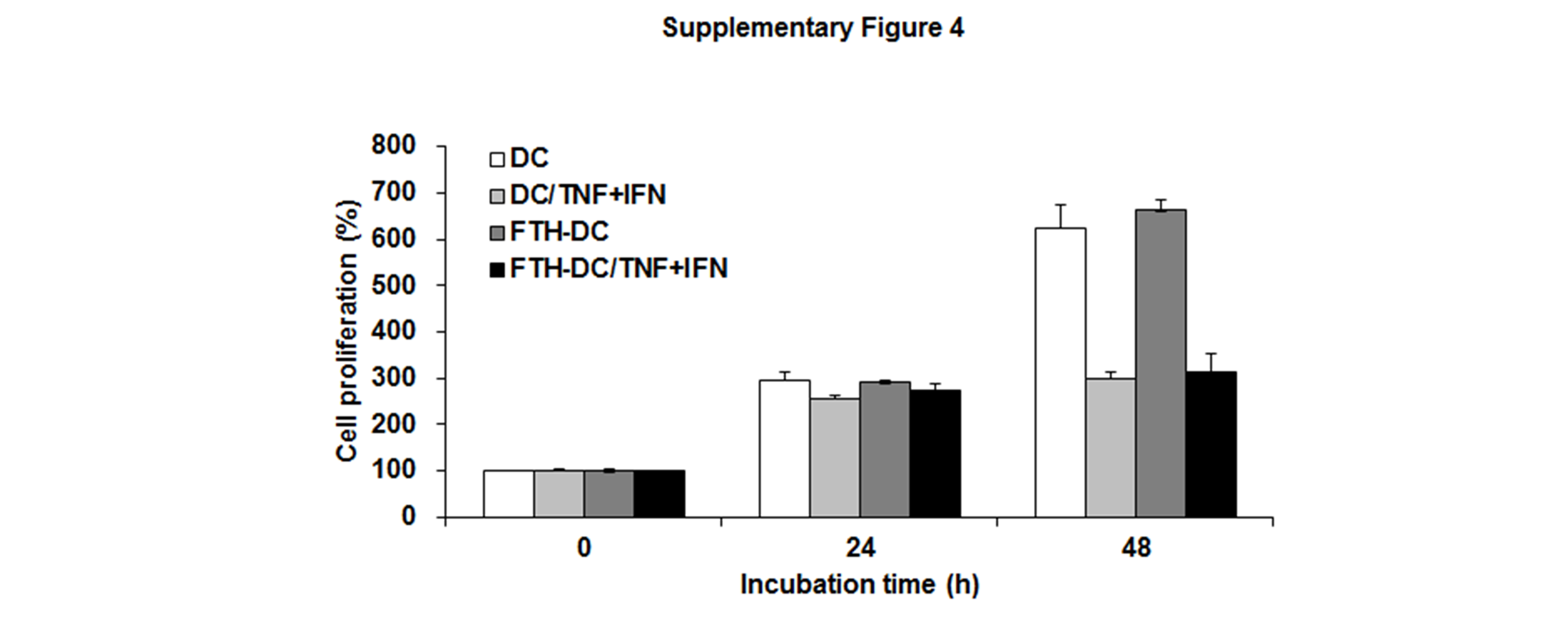

Supplement: S4 Fig — A standard 3-,5-diphenyltetrazolium bromide (MTT) assay for proliferation activity of DCs and FTH-DCs stimulated by with TNF-α (20 ng/mL) and IFN-γ (20 ng/mL) for 24 h and 48 h. (TIF) [file pone.0125291.s004.tif]

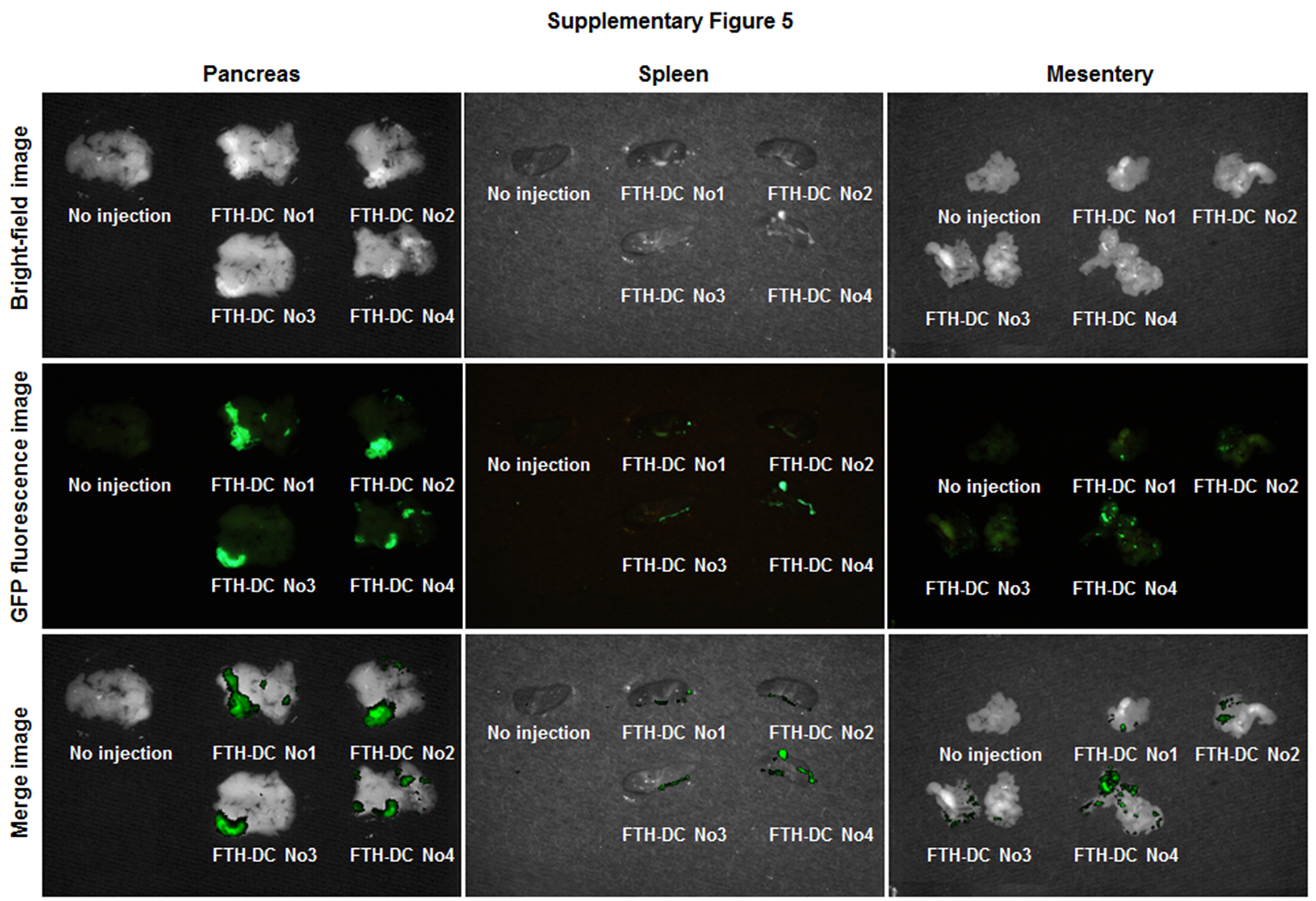

Supplement: S5 Fig — 6-7-week old mice (n = 5) were intraperitoneally injected with 1x10^7^ FTH-DCs incubated in the medium including TNF-α (20 ng/mL) and IFN-γ (20 ng/mL) for 24 h. A total of 1 x 10^7^ FTH-DCs were injected subcutaneously into the hind footpads of mice. At 3 and 5 days after intraperitoneal injection of FTH-DCs, GFP fluorescence can be observed in isolated pancreas, mesentery and spleen using the Maestro fluorescence imaging system. These results demonstrated that FTH-DCs still migrated to other organs and reached at LN in the pancreas or the mesentery as well as the spleen, which is largest lymphatic organ. (TIF) [file pone.0125291.s005.tif]
